# House dust mite sensitisation and association with atopic dermatitis in Brunei: Allergen sensitization and allergic disease in Brunei

**DOI:** 10.1186/s13601-019-0304-5

**Published:** 2019-12-23

**Authors:** Haziq Emran, Christina Siew Eng Chieng, Surita Taib, Anne Catherine Cunningham

**Affiliations:** 1 Pengiran Anak Puteri Rashidah Sa’adatul Bolkiah (PAPRSB), Institute of Health Sciences, Universiti Brunei Darussalam, Gadong, Brunei Darussalam; 2 Raja Isteri Pengiran Anak Saleha (RIPAS) Hospital, Bandar Seri Begawan, Brunei Darussalam

**Keywords:** Dust mite allergy, Atopic dermatitis, Allergens, Atopy

## Abstract

IgE sensitisation in tropical areas is under-reported. A 2 year retrospective cohort study of allergy data specific to aero and food allergens in Brunei demonstrated that specific IgE levels to house dust mite (*Dermatophagoides pteronyssinus, D.farinae, Blomia tropicalis*) were highest in this population and correlated with atopic dermatitis (*p* < 0.001). Shrimp and peanut were the most common food allergens. A dominance of house/storage mite sensitization is seen in Brunei which is consistent with other tropical countries.

## Background

Allergy is on the increase, and there are differences in the development of allergic disease dependent on climate, diet and lifestyle. Allergy may present as respiratory symptoms (e.g. dyspnoea, rhinitis), skin manifestations (e.g. pruritus, atopic dermatitis) or others (e.g. angioedema, urticaria). House dust mite is a potent allergen and sensitisation can lead to asthma, rhinitis, conjunctivitis and atopic dermatitis [1]. Strategies to reduce house dust mite exposure in early childhood have been effective in reducing morbidity associated with allergic diseases and medication requirements.

In Brunei, the number of inpatient and outpatient submissions with asthma and related respiratory symptoms increased from 2538 patients per 100,000 in 2005 to 2935 patients per 100,000 in 2010 equalling to about 15% increment over a 5-year period. In 2017, asthma was the 5th cause of in-patient morbidity (1256 patients; 298.1 per 100,000 of the population) [2].

Sensitisation to food allergens varies according to age, climate, geography and genetic background. Atopic dermatitis is often associated with but not limited to food allergy. It can also be a manifestation of a skin allergy to house dust mite [1].

## Methods

A retrospective cohort study was performed on all patients subjected to specific IgE blood tests (sIgE) in 2015 and 2016. The data (demographic, patient history, total and specific IgE levels) was collected anonymously from the patients’ electronic record (BruHIMS) with no further sampling or exclusion criteria. All data collection was done with permission of RIPAS Hospital and approved by the Medical and Health Research Ethics Committee, Ministry of Health (MHREC).

Specific IgE levels to a panel of defined inhaled allergens were measured in patient serum by fluoroenzymeimmunoassay using the Phadia 100 platform. Serum ≥ 0.10 kUA/L was considered to be positive for specific IgE tests (sIgE) and Serum ≥ 100 kUIgE/L was considered positive for total IgE tests (tIgE).

IBM SPSS Statistics v21 was used to analyse data with *p *< 0.05 considered statistically significant. Graphpad Prism v8.1.2 was used to plot data.

### Spectrum of allergen sensitisation

**Fig. 1:**
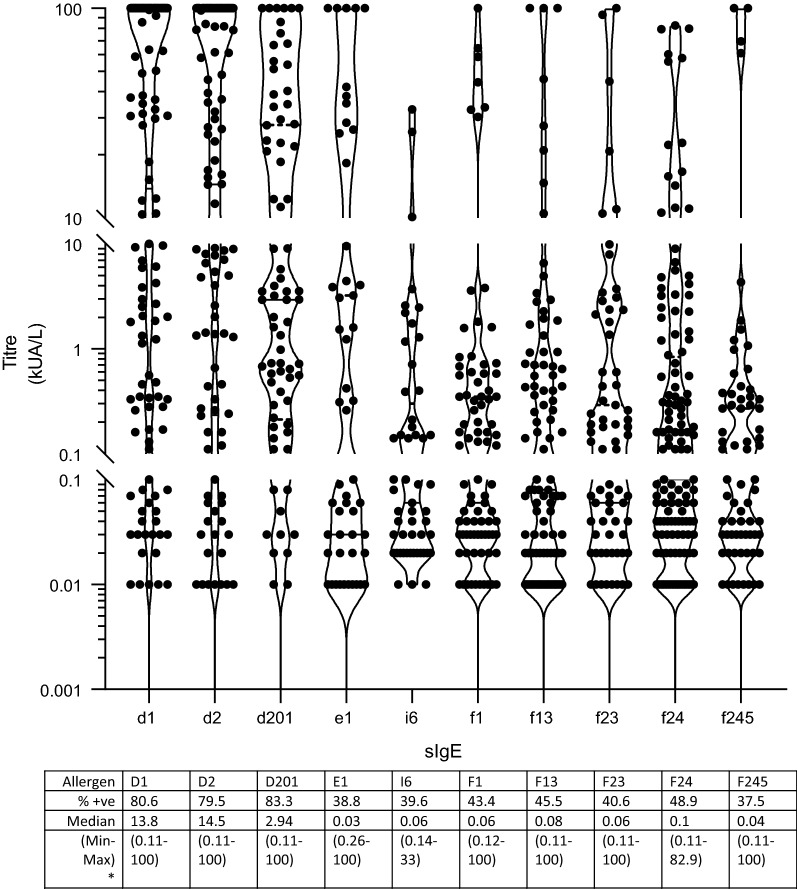
Levels of specific IgE titres to the top 5 aero and food allergens measured (D1 *D. Pteronyssinus*, D2 *D. farinae*, D201 *B. tropicalis*, E1 Cat dander, I6 German cockroach; F1 Egg White, F13 Peanut, F23 Crab, F24 Shrimp, F245 Egg) *min–max values for positive individuals

One hundred and seventy patients had at least one positive sIgE (out of 223 patients tested). The mean age of patients was 27.8 (SD = 18.05 median 25 range 0–80), including 99 (44.4%) males and 124 (55.6%) females. The frequency of positive sIgE was highest in younger patients, peaking in the 16–20 year age group. Most patients were Malay (74.1%) followed by Chinese (19.3%). The rest (4.2%) were indigenous (e.g. Iban), expatriate (e.g. Philipino, Nepalese) or Indian (2.4%). The majority of samples came from Dermatology (73%), followed by Oral Maxillo-Facial Department (16%). Eleven percent came from other departments (General Internal Medicine, Cardiology, Palliative, Hematology, Pediatrics, General Surgery and General ward). The most prevalent aeroallergen was *Dermatophagoides pteronyssinus* (d1; *n *= 100 positive results) followed by *D. farinae* (d2; *n *= 89) and *Blomia tropicalis* (d201; *n *= 65). Shrimp (f24; *n *= 67) was the most common food allergen, followed by peanut (f13; *n *= 45) and egg white (f1; *n *= 43). Total IgE significantly correlated with sIgE *p *< 0.001. Figure 1 illustrates sIgE titres across the top 5 aero and food allergens measured.

### Association between allergen sensitisation and common allergic diseases

**Fig. 2:**
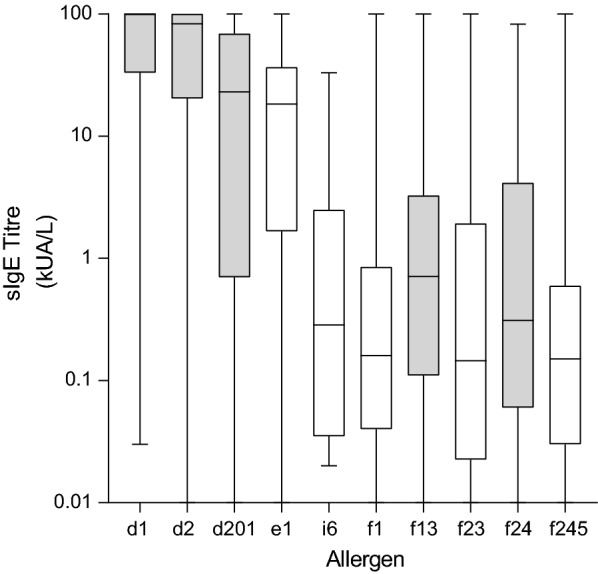
Boxplot (min to max) representing sIgE titres to the most common aero and food allergens in Atopic Dermatitis patients (*n* = 89) (D1 *D. Pteronyssinus*, D2 *D. farinae*, D201 *B. tropicalis*, E1 Cat dander, I6 German cockroach; F1 Egg White, F13 Peanut, F23 Crab, F24 Shrimp, F245 Egg). Gray bars represent a significant association (*p* > 0.001)

Atopic Dermatitis was associated with sensitisation to all 5 most common allergens (D1 *D. pteronyssinus*, D2 *D. farinae*, D201 *B. tropicalis*, F13 Peanut, F24 Shrimp all *p* < 0.001). The levels of sIgE to the most common aeroallergens and food allergens in atopic dermatitis patients are illustrated in Fig. 2. There was no significant association between sensitisation to any allergen and any other allergic diseases including eczema, urticaria, asthma or rhinitis in this patient population (*p* > 0.05).

It is apparent that there is a very high sensitisation to house dust mite species (e.g. *D. pteronyssinus, D. farina, B. tropicalis*). Atopic dermatitis appears to be predominantly a skin manifestation of allergy to house dust mite in Brunei. There was also a statistically significant association with sIgE to shrimp and peanut, both common in the local diet.

Brunei is a tropical environment with high humidity. House dust mite is the dominant allergen, which is similar to other urban tropical environments (e.g. Singapore) where a dominant mono-specific sensitization to house dust mite was also linked to the burden of allergic disease [3, 4] A predominance of house dust mite sensitisation has also been seen in the Philippines (via sIgE), Thailand (via skin prick testing) [5, 6] and South Korea [7, 8]. The climate favours the survival and growth of house dust mites which may lead to increased exposure and sensitization in tropical regions, including Brunei.

It was surprising that there were relatively few asthmatic patients referred for sIgE testing. It would be expected that asthmatic patients would be sensitized to house dust mite. An earlier study from Brunei published in 1980 demonstrated that 40/60 asthmatics tested at the same hospital had a positive skin prick test to *D. pteronyssinus,* d1 and 37/60 to *D. farinae* d2 [9], the two allergens which showed the highest levels of sIgE and prevalence of sensitisation in this study.

Most patients referred allergy testing came from dermatology, 89 were diagnosed with atopic dermatitis and 16 of these patients also had asthma. Education to raise awareness of reducing house dust mite growth could help reduce sensitization and response to environmental allergens, particularly *D. pteronyssinus* (d1), *D. farinae* (d2) and *B. tropicalis* (d201).

There is limited data on food allergy in tropical countries. Brunei’s profile was dominated by shrimp allergy which matches the international trend. There may be some may be some cross reactivity between shrimp and *D. pteronyssinus* allergens, in particular tropomyosin which is a pan allergen across arthropods including arachnids e.g. mites, insects e.g. cockroach and crustaceans e.g. shrimp [1]. Further studies using recombinant Der p 10 allergen would be needed to confirm this.

Specific IgE and total IgE are positively associated with each other. Total IgE can be used as a screening tool before following up on requests for sIgE. Future studies should investigate tIgE and sIgE to house dust mite in asthmatic patients, given the increasing prevalence of this disease in Brunei.

## Data Availability

This data will belongs to the Ministry of Health, Brunei Darussalam (as per Ethics Approval). I can request this data to be made available according to your publication policy.

## References

[1] Miller JD (2018). The role of dust mites in allergy. Clin Rev Allergy Immunol.

[2] Health information booklet 2017. updated January 2019, Ministry of Health: Brunei Darussalam. http://www.moh.gov.bn/SitePages/Health%20Information%20Booklet.aspx Accessed 4 July 2019.

[3] Andiappan AK (2014). Allergic airway diseases in a tropical urban environment are driven by dominant mono-specific sensitization against house dust mites. Allergy.

[4] Andiappan AK (2014). Allergic airway diseases in a tropical urban environment are driven by dominant mono-specific sensitization against house dust mites. Eur J Allergy Clin Immunol.

[5] Daengsuwan T (2003). Allergen Sensitization to aeroallergens including blomia tropicalis among adult and childhood asthmatics in thailand. Asian Pacific J Allergy Immunol.

[6] Yap JMG (2014). Multiple house dust mite allergen- sensitization profiles in children with allergic asthma. J Allergy Ther.

[7] Kim HS (2015). Immunoglobulin E to allergen components of house dust mite in Korean children with allergic disease. Asia Pac Allergy.

[8] Jeong KY (2015). Profiles of IgE sensitization to Der f 1, Der f 2, Der f 6, Der f 8, Der f 10, and Der f 20 in Korean house dust mite allergy patients. Allergy Asthma Immunol Res.

[9] Woodcock AA, Cunnington AM (1980). The allergenic importance of house dust and storage mites in asthmatics in Brunei, S.E Asia. Clin Exp Allergy.

